# Health Benefits of Bioactive Compounds from the Genus *Ilex*, a Source of Traditional Caffeinated Beverages

**DOI:** 10.3390/nu10111682

**Published:** 2018-11-05

**Authors:** Ren-You Gan, Dan Zhang, Min Wang, Harold Corke

**Affiliations:** Department of Food Science & Technology, School of Agriculture and Biology, Shanghai Jiao Tong University, Shanghai 200240, China; renyougan@sjtu.edu.cn (R.-Y.G.); zhang.dan@sjtu.edu.cn (D.Z.); wangmin799@outlook.com (M.W.)

**Keywords:** kudingcha, yerba mate, yaupon holly, guayusa, caffeine, polyphenols

## Abstract

Tea and coffee are caffeinated beverages commonly consumed around the world in daily life. Tea from *Camellia sinensis* is widely available and is a good source of caffeine and other bioactive compounds (e.g., polyphenols and carotenoids). Other tea-like beverages, such as those from the genus *Ilex*, the large-leaved Kudingcha (*Ilex latifolia* Thunb and *Ilex kudingcha* C.J. Tseng), Yerba Mate (*Ilex paraguariensis* A. St.-Hil), Yaupon Holly (*Ilex vomitoria*), and Guayusa (*Ilex guayusa* Loes) are also traditional drinks, with lesser overall usage, but have attracted much recent attention and have been subjected to further study. This review summarizes the distribution, composition, and health benefits of caffeinated beverages from the genus *Ilex*. Plants of this genus mainly contain polyphenols and alkaloids, and show diverse health benefits, which, as well as supporting their further popularization as beverages, may also lead to potential applications in the pharmaceutical or nutraceutical industries.

## 1. Introduction

Caffeine (1,3,7-trimethylxanthine) is a member of a group of compounds known as purine alkaloids [1], occurs naturally in plants used to make beverages such as coffee and tea, and is added in the formulation of many soft drinks. Caffeine is a well-known central nervous system stimulant in humans. Tea from *Camellia sinensis* is the most popular non-alcoholic caffeine-containing beverage, and has a long consumption history all over the world. Major chemical constituents in tea are polyphenols, proteins, enzymes, caffeine, carbohydrates, and inorganics, which provide health beneficial properties [1,2]. However, caffeinated tea-like beverages with somewhat comparable chemical characteristics are also obtained from plants of the genus *Ilex*, mainly the large-leaved Kudingcha, Yerba Mate, Yaupon tea, and Guayusa tea, which have been well studied in recent years. The genus *Ilex*, comprising some 600 species, is widely distributed across most non-tropical parts of the world. The best-known species in Western literature is the European or English Holly, *I. aquifolium* L., with its characteristic red drupes (berries) and leaves widely used in Christmas decorations.

Kudingcha has a long consumption history in China and its commercial products are commonly found in the market. The large-leaved Kudingcha, including *Ilex latifolia* Thunb and *Ilex kudingcha* C.J. Tseng, have been reported to show significant medicinal or bioactive properties such as antioxidant, anti-inflammatory, anti-obesity, anti-cancer, modulation of gut microbiota, and antiproliferative effects [3,4,5,6,7,8,9,10]. In addition, Yerba Mate produced from leaves of the tree *Ilex paraguariensis* is a widely consumed beverage in South American countries such as Argentina, Brazil, Chile, Paraguay, and Uruguay, and the average annual consumption reaches around 3 kg to 10 kg per person. Yerba Mate tea has developed into a main alternative to coffee and black tea since it is characterized as having various health benefits, such as antimicrobial, antioxidant, anti-obesity, anti-diabetic, and cardiovascular protective effects [11,12,13,14,15,16,17,18]. Moreover, in the southeastern part of the United States, Yaupon tea (Yaupon Holly, *Ilex vomitoria*) is prepared as a healthy beverage by Native Americans [19]. The polyphenolics extracted from Yaupon Holly are free of catechin, and exhibit antioxidant, anti-inflammatory, and chemo-preventive effects [19,20,21]. Compared to green tea, processing and packaging have less effect on the degradation of polyphenolics in Yaupon Holly, indicating an advantage for commercial products of Yaupon tea. Guayusa tea, commercially known as Runa tea, is natively grown in the Amazon and has long been consumed by Amazonian indigenous tribes [22,23]. *Ilex guayusa* tea contains high levels of phenolic compounds, a good dietary resource with cellular antioxidant and anti-inflammatory properties [23,24].

Therefore, in order to provide a better understanding of *Ilex*-based caffeinated beverages, the relevant literature from the last ten years was searched in Web of Science. The geographical distributions of different *Ilex* species are summarized, followed by a discussion of their main bioactive compounds, and finally we highlight the potential health benefits and related molecular mechanisms. Since many *Ilex* species are already commonly consumed in the world, the information in this review will help to provide a scientific structure to explain the health benefits of *Ilex*-based beverages, which may encourage further development by the *Ilex* tea industry and lead to new products for the public.

## 2. Distribution

Plants of the genus *Ilex* are distributed widely in various parts of the world (Table 1). Large-leaved Kudingcha, an infusion made from evergreen trees of two species (*I. kudingcha* C.J. Tseng and *I. latifolia* Thunb.), is a popular bitter-tasting infused tea found in China and other Southeastern Asian countries (e.g., Singapore, Malaysia, and Vietnam) [3,25]. Yerba Mate tea, from a native South American holly shrub, is mainly produced and consumed in South America [26,27]. A study from Marcelo et al. reported that Yerba Mate could possibly be identified as to country of origin in South America by elemental concentration and chemometrics [28]. Leaves of Yaupon Holly (*Ilex vomitoria*), from an evergreen and caffeine-containing shrub native to the southeastern United States, was used to make a healthy beverage by Amerindians and later European colonists [19,29]. Guayusa is made from leaves of an evergreen tree native to South America and is grown in the Amazon. Guayusa has recently gained more attention [22,23].

## 3. Bioactive Compounds

*Ilex* genus plants are generally known to be rich in a wide variety of bioactive compounds, mainly polyphenols and alkaloids, which play an essential role in their health benefits.

### 3.1. Polyphenols

#### 3.1.1. Polyphenols in Large-Leaved Kudingcha

Structurally, polyphenols are a class of compounds composed of benzene rings bonded to one or more hydroxyl groups. In previous published studies, different methods have been applied to determine the phenolic composition in Kudingcha. For example, the use of tyrosinase biosensor, Folin-Ciocalteu assay, high performance liquid chromatography (HPLC), HPLC-nuclear magnetic resonance (NMR), ultra-high performance liquid chromatography (UHPLC), UHPLC-diode array detector-linear ion trap-Orbitrap (UHPLC-DAD-LTQ-Orbitrap), liquid chromatography-photodiode array detector-atmospheric pressure chemical ionization-mass spectrometry (LC-PDA–APCI-MS), and the quantitative analysis of multiple components with a single marker (QAMS) methods were reported [3,24,25,30,37]. Using these methods, polyphenols can be identified and quantified effectively.

**Figure 1:**
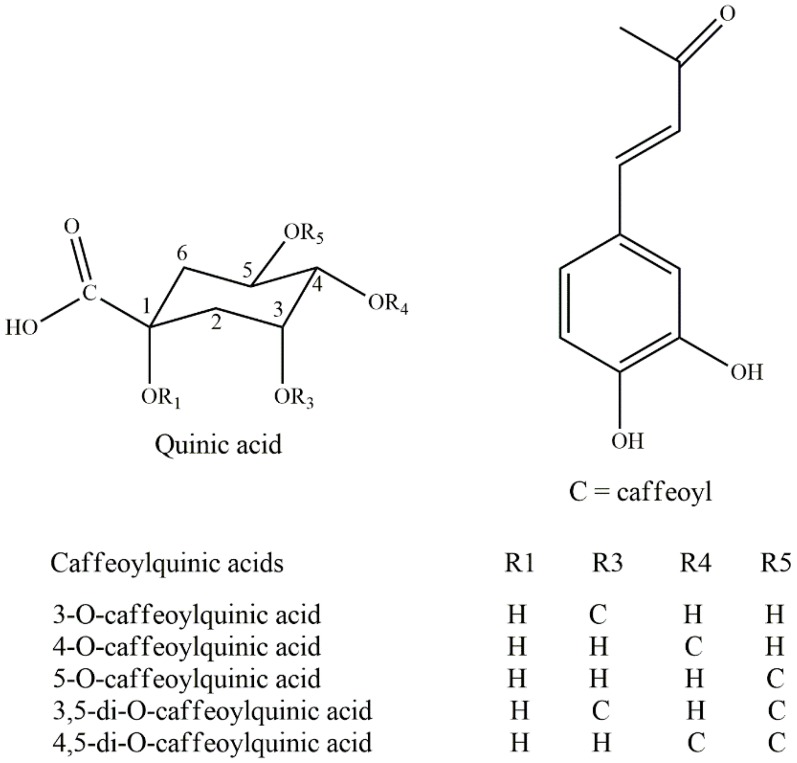
The chemical structures of caffeoylquinic acids.

The total polyphenolic content (TPC) in *I. latifolia* was 188 mg gallic acid equivalent (GAE) per g dry plant material using the Folin–Ciocalteu method [10]. Caffeoylquinic acids (Figure 1) and their derivatives are the main polyphenols in Kudingcha. Compounds such as ethyl caffeate, 3,4-di-*O*-caffeoylquinic acid methyl ester, 3,5-di-*O*-caffeoylquinic acid methyl ester, and chlorogenic acid were identified in *I. latifolia* [38]. Chlorogenic acid (CGA), the ester of caffeic acid and quinic acid, is known for its biological functionality. The CGA derivatives 3-*O*-caffeoylquinic acid, 5-*O*-caffeoylquinic acid, 3,5-*O*-dicaffeoylquinic acid, and 4,5-*O*-dicaffeoylquinic acid have been identified as major compounds in methanol and ether acetate extracts of *I. kudingcha* [39]. This was confirmed by Che et al., who detected a total of 68 CGA candidates belonging to 12 categories [40]. Our previous study also reported that isomers of mono- and di-caffeoylquinic acids were the predominant compounds from Kudingcha genotypes of two *Ilex* species, and the average amount of major CGAs from these Kudingcha of different origins was 97 mg/g [25]. Furthermore, 18 active components including polyphenols, such as hydroxycasein, protocatechuic acid, rutin, neochlorogenic acid, chlorogenic acid, cryptochlorogenic acid, caffeic acid, and isochlorogenic acid, were first determined in *I. kudingcha* by the QAMS method, which was efficiently applied for simultaneous determination of different phenolic compounds [30]. Moreover, three caffeoylquinic acids, including neochlorogenic, chlorogenic, and cryptochlorogenic acids, and three dicaffeoylquinic acids, were identified as the main constituents in *I. kudingcha* [37].

#### 3.1.2. Polyphenols in Yerba Mate

Polyphenols have been extracted from different parts of Yerba Mate, such as the whole plant, leaves, and stems. Of these, the highest level of phenolic compounds was found in the leaf extract [15]. From chromatographic analyses, the TPC was determined as about 51 mg/g dry mass (DM) in *I. paraguariensis* [41]. Moreover, determination of the TPC in Yerba Mate was performed by the Folin-Ciocalteu method, where 111 samples from the Parana State in Brazil were characterized [18]. Additionally, 46 different polyphenols from four commercial Yerba Mate products have been quantified, with hydroxycinnamic acid derivatives and flavonols accounting for 90% and 10% of the polyphenols present. Of these, 3-caffeoylquinic (26.8% to 28.8%), 5-caffeoylquinic (21.1% to 22.4%), 4-caffeoylquinic (12.6% to 14.2%), and 3,5-dicaffeoylquinic acids (9.5% to 11.3%) along with rutin (7.1% to 7.8%) were found to be the predominant polyphenolic compounds. In conclusion, *I. paraguariensis* was shown to be a good source of polyphenols [18,27]. Moreover, the content of lutein in aqueous extracts of Yerba Mate varied in different commercial samples, giving further prospects for a role in risk reduction for certain diseases [42].

#### 3.1.3. Polyphenols in Yaupon Holly

Recently, limited research has been carried out on Yaupon Holly (*I. vomitoria*). In infusions, eight polyphenolic compounds were identified including mono-caffeoylquinic acids, di-caffeoylquinic acids, and two flavonol glycosides (quercetin 3-rutinosides and kaempferol 3-rutinoside), where the mono- and di-caffeoylquinic acids comprised 70% of the total polyphenolics [20]. Kim and Talcott also determined the composition of diverse polyphenolic compounds in tea infusion of Yaupon Holly, and found that 3-*O*-caffeoylquinic acid (chlorogenic acid), quercetin 3-rutinoside (rutin), 5-*O*-caffeoylquinic acid (neochlorogenic acid), and 4-*O*-caffeoylquinic acid (cryptochlorogenic acid) were the main phenolic compounds, with 423, 392, 318, and 125 mg/L rutin equivalents, respectively [21].

#### 3.1.4. Polyphenols in Guayusa

*I. guayusa* teas showed high polyphenolic content totaling between 54 and 67 mg GAE/g DM, and phenolic mono- and di-caffeoylquinic acid derivatives were the major compounds determined by mass spectrometry [23]. Moreover, determination of TPC by the Slinkard and Singleton method showed a very different content in green leaves and in processed Guayusa [22]. For green leaves, TPC was about 55 mg/g DM, with hydroxycinnamic acid derivatives as the major constituents. The levels of 5-*O*-CQA (chlorogenic acid), 3,5-Dicaffeoylquinic acid (isochlorogenic acid), and 3-*O*-CQA (neochlorogenic acid) were 24, 16, and 8 mg/g DM, respectively. Processing methods such as blanching and fermentation are important factors affecting the TPC in Guayusa [22]. Kapp et al. reported that catechin, epicatechin, epicatechin gallate, epigallocatechin, and epigallocatechin gallate (EGCG) were found in *I. guayusa* leaves [36]. Other research showed that the major constituents of phenolics were hydroxycinnamic acid, and chlorogenic acid was the main phenolic compound found in both young and old leaves of Guayusa [24].

Overall, caffeoylquinic acids and their derivatives are the main phenolic compounds in the genus *Ilex*, which are summarized in Table 2.

### 3.2. Alkaloids

#### 3.2.1. Alkaloids in Large-Leaved Kudingcha

**Figure 2:**
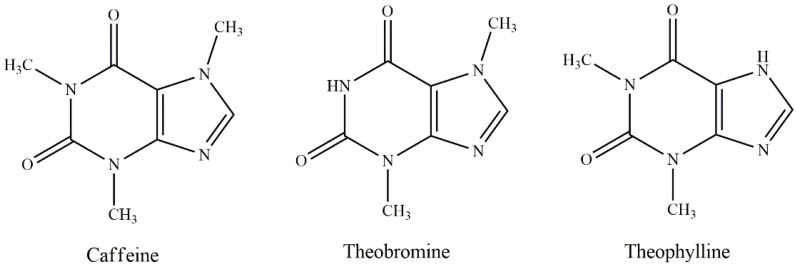
The chemical structures of main alkaloids in the genus *Ilex*.

Alkaloids are a class of naturally occurring compounds that mostly contain basic nitrogen atoms, showing a wide range of physiological and pharmacological effects. Methylxanthines, mainly caffeine and theobromine (Figure 2), are the main alkaloids in large-leaved kudingcha. However, alkaloid content is relatively low. It was reported that the content of total methylxanthines was around 7% to 9%, of which caffeine accounted for 3% to 6%, while theobromines made up only 0.1% [1].

#### 3.2.2. Alkaloids in Yerba Mate

Methylxanthines are alkaloids naturally present in Yerba Mate, mainly comprising caffeine and theobromine [18,27,31]. It was reported that near infrared spectroscopy analysis could be applied to predict the total methylxanthine content in Yerba Mate. The total amount of methylxanthine in 25 samples of Yerba Mate ranged from 3.69 to 12.7 mg/g, with concentrations of caffeine and theobromine as 0.001 to 10.1 and 0.02 to 5.03 mg/g, respectively [31]. To quantify theobromine and caffeine in *I. paraguariensis* extracts, quality by design (QbD) models and UHPLC were optimized and applied, and indicated good future potential for application of this methodology [32]. In another study of samples of Yerba Mate methylxanthines were quantified by HPLC–DAD, with caffeine consistently higher in content than theobromine. Overall, Yerba Mate, with the total methylxanthines ranging from 8.2 to 10.2 mg/g, can be regarded as a moderate source of these purine alkaloids [18].

#### 3.2.3. Alkaloids in Yaupon Holly

To identify residues of caffeinated beverages, three xanthines theobromine, theophylline, and caffeine (Figure 2) are commonly used as standards. It was found that Yaupon beverages contained all three, but their concentrations were significantly different between wild and domesticated types [34]. Moreover, the caffeine content in dioecious Yaupon Holly was 0% to 1.91% of dry weight, with the level strongly affected by nitrogen fertilizer but not by gender [19,29]. In another study, caffeine was undetectable by HPLC in Yaupon Holly leaves [43].

#### 3.2.4. Alkaloids in Guayusa

The tea of *I. guayusa,* prepared by steeping leaves in boiling water, is consumed by Amazonian families and has a high caffeine content [23]. Kapp et al. found that the extract of Guayusa contained several secondary metabolites, such as caffeine and theobromine, at 36 and 0.3 mg/mL, respectively [36]. Extracts from *I. guayusa* have also been shown to contain caffeine [23].

## 4. Health Benefits

**Figure 3:**
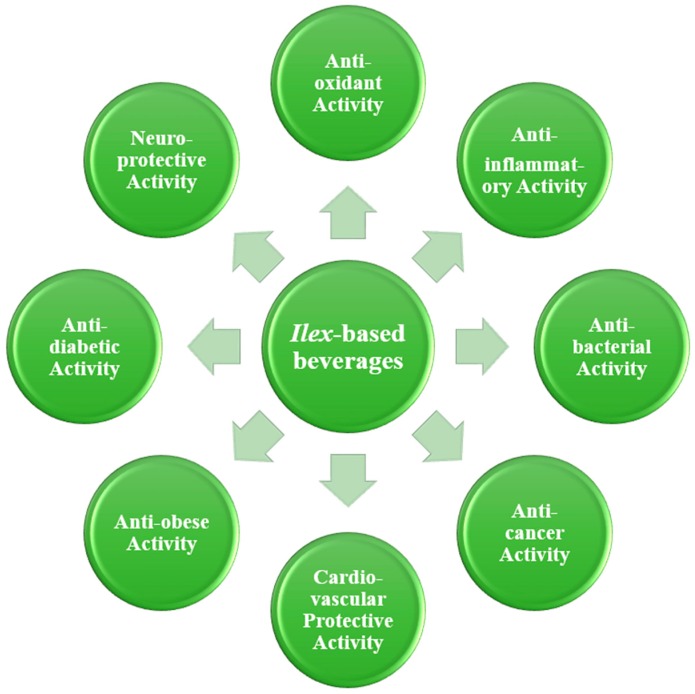
The health benefits of caffeinated beverages from the genus *Ilex*.

Some of the physiological effects of caffeinated beverages from *Ilex* are potentially beneficial for human health (Figure 3). Here, we further discuss the actions and related mechanisms of these potential benefits from different *Ilex* species.

### 4.1. Antioxidant Activity

Consumption of herbal teas prepared from *I. paraguariensis*, *I. vomitoria*, *I. kudingcha*, and *I. guayusa* have been reported to exhibit high reducing power, 2,2-diphenyl-1-picrylhydrazyl (DPPH) scavenging and lipid peroxidation inhibition activities, thus relieving oxidative damage [44]. Based on the in vitro ferric-reducing antioxidant power (FRAP) assay, one extracted alkaloid constituent from *I. latifolia* had high reducing power [5]. Our previous study also found that the methanol extracts of six Kudingcha genotypes of the genus *Ilex* had relatively high in vitro antioxidant activity based on different antioxidant assays [25]. *I. guayusa* tea aqueous extracts (1 g/mL) prepared by conventional means protected 70% to 80% Caco-2 cells from oxidative damage [23], and prevented lipid peroxidation and DNA oxidative damage induced by ultraviolet radiation [45]. Similar antioxidant ability was also found in vivo and in human studies. Pereira et al. found that giving a gavage of Mate tea (20 mg/kg BW/day) to female Wistar rats minimized oxidative stress induced by hormonal changes during perimenopause [46]. Moreover, the impaired endogenous antioxidant defense system in the host could also be recovered by these widely consumed non-alcoholic beverages. As reported, the long-term ingestion of Mate tea (1 L/day) contributed to the increase in ferric-reducing antioxidant potential in dyslipidemic subjects [44], as well as the increased glutathione (GSH) concentration and decreased serum lipid hydroperoxides (LOOH) levels in type 2 diabetic mellitus (T2DM) subjects [47]. In addition, the acute consumption of freeze concentrated Yerba Mate infusion (100 mL) also enhanced the activities of antioxidant enzymes in healthy individuals, including catalase (CAT, 28.7%), superoxide dismutase (SOD, 21.3%), and glutathione peroxidase (GPx, 9.6%) in blood samples [48].

It is widely accepted that the counteraction on oxidative stress is mainly attributable to the existing phenolic compounds, especially chlorogenic acids (like mono- and dicaffeoylquinic acids) as well as flavonols [49,50], since in vitro antioxidant capacity has been confirmed to be positively correlated with their concentrations [13,51]. In order to better retain the contents and stability of health-beneficial antioxidants in *Ilex* teas, more attention should be paid to the adjustment of industrial processing methods and the improvement of packaging methods [22,52].

### 4.2. Anti-Inflammatory Activity

The inflammatory response is usually accompanied by the activation of macrophages, neutrophils, and various released inflammatory cytokines, such as tumor necrosis factor-α (TNF-α), interleukin (IL)-1β, -6, and -12, triggering histological damage to specific tissues. Reduction of the exudate concentration, reestablishment of the balance between pro- and anti-inflammatory cytokines (IL-4 and -10), and suppression of the pro-inflammatory enzyme activity are considered to be major therapeutic targets in inflammation treatment.

High anti-inflammatory effects were observed in RAW 264.7 cells treated with *I. latifolia* ethanol extract (50 μg/mL), coupled with reduced nitric oxide (NO) production, which could dilate small blood vessels and increase the infiltration of pro-inflammatory mediators [5,10]. Similarly, a 10% to 30% NO inhibition rate was also reported in *I. guayusa* aqueous extracts (1 g/mL) treatment [23]. In addition, several animal experiments also reported the anti-inflammatory effects of these tea-like beverages. For instance, *I. kudingcha* C. J. Tseng methanol extracts (KME) administration upregulated the mRNA expression of inducible nitric oxide synthase (iNOS) and reduced the formation of pro-inflammatory factors like TNF-*α*, IL-1*β*, and IL-6 in dextran sulfate sodium (DSS)-induced ulcerative colitis (UC) mice [53]. Besides, *I. paraguariensis* has been reported to show anti-inflammatory effects in various animal models, such as pleurisy in mice [54], cigarette smoke-induced acute lung inflammation in mice [55], obesity-related inflammation in rats [56,57], azoxymethane-induced inflammation in a rat colon [58], and acute edema in a mouse model [59] at concentrations ranging from 150 mg/kg to 250 mg/kg. The anti-inflammatory mechanism of Yerba Mate was reported to inhibit the NF-*κ*B signaling pathway through restraining the phosphorylation of upstream IκB-*α* and GSK-3*β*, leading to blocking downstream iNOS and cyclooxygenase-2 (COX-2) expression, and the secretion of inflammatory cytokines [56,58]. However, the anti-inflammatory effects observed in several animal models have not been reported in human studies. Preliminary evidence has shown that Yerba Mate consumption (3 g Yerba Mate diluted in 200 mL water once a day for sixty days) did not alter the inflammatory parameters like high-sensitivity C-reactive protein (hs-CRP), fibrinogen, and HDL-C levels in 92 HIV/AIDS-positive individuals. The discrepancy between basic research and clinical cases could be due to the amount of beverage offered, the concentration of bioactive compounds in Mate tea, and the metabolic conditions of specific populations [60].

### 4.3. Antibacterial Activity

Compared to tea from *Camellia sinensis*, research on the antibacterial properties of Yerba Mate is relatively limited [27]. The antibacterial effects of Yerba Mate have been reported for *Escherichia (E.) coli*, *Salmonella typhimurium*, *Listeria monocytogenes*, *Staphylococcus (S.) aureus*, and even methicillin-resistant *S. aureus* (MRSA), with the antibacterial concentration ranging from 40 μg/mL to 7.4 mg/mL, and the inhibitory effects seemingly better on gram-positive bacteria than gram-negative bacteria [61]. Commonly, higher concentrations are required when applied to food systems due to the interaction of antibacterial substances with food components like proteins and lipids. Burris et al. found that the concentration of lyophilized aqueous extract of Yerba Mate in apple juice (40 mg/mL) was eight-fold higher than that in a medium (5 mg/mL) for equivalent bacterial inactivation [62]. A similar conclusion was also reached for ground beef, where the anti-MRSA concentration of Mate tea increased dose-dependently with increase of fat content [63].

Although the composition of Yerba Mate extract is relatively clear, conflicting results are shown with regards to the identification of bioactive compounds responsible for antimicrobial activity. Apart from the generally believed phenolic compounds [64], 3,4-dihydroxybenzaldehyde could significantly inhibit MRSA growth even at the lowest concentration of 100 μg/mL [65]. Besides, macromolecules like protein, occupying about 26% of Yerba Mate, might be responsible for the antibacterial activity since dialyzed aqueous extracts have also shown inhibitory effects on *E. coli* and *S. aureus* [62]. The antibacterial mechanism has been much less investigated, and it was pointed out that the tea extract of *I. paraguariensis* had a destructive effect on the central carbon metabolism and energy production pathways, as well as cell membrane integrity [66]. Overall, it is still unclear whether the ingredients that have important antibacterial properties are completely identified and whether they have synergistic or additive antibacterial effects.

### 4.4. Lipid-Reducing Activity

Several in vitro, in vivo, and human studies have reported the lipid-lowering benefits of the extract of *I. paraguariensis*. The inhibited accumulation of triglycerides in HepG2 cells and attenuated blood lipid levels were demonstrated in *I. latifolia* aqueous extracts [7,67]. Besides, *N*-butanolic fraction (n-BFIP), a standardized fraction rich in phenolic compounds derived from Yerba Mate was also shown to reduce triglycerides (TG) and low-density lipoprotein cholesterol (LDL-C) in high-fat-diet induced (HFD) rats by 30% and 26%, respectively [68]. This was consistent with the conclusion that polyphenols and methylxanthines in Yerba Mate showed higher lipid-reducing activity than saponins [69]. In addition, the lipid-reducing effect of Yerba Mate extract was proven to be effective not only in animal models, such as hyperlipidemic hamster model [70], rats [68], and rabbits [69], but also in humans. Dyslipidemic and normolipidemic subjects supplemented with 50 g (330 mL infusion and 3 times/day) Yerba Mate had about 10% reduction in lipid parameters (LDL-C and TG) [71,72]. In addition, it was reported that heavy drinkers of *I. paraguariensis* beverage (>1 L/day) had lower total cholesterol, LDL-C, and fasting glucose, but interestingly, their body weight was higher, compared with moderate drinkers [73]. This low-lipids high-body-weight paradox observed in the population of heavy drinkers of *I. paraguariensis* beverages could be due to the induced hypoglycemia and compensatory higher intake of refined carbohydrates, since their consumption of carbohydrates was higher than moderate drinkers [73].

For the lipid-reducing molecular mechanism, triterpenoid saponins (200 mg/kg/day) derived from *I. latifolia* was reported to lower lipids by the inhibition of sterol regulatory element-binding proteins (SREBPs) via enhancing AMP-activated protein kinase (AMPK) phosphorylation in a non-alcoholic fatty liver disease mouse model [74,75]. In addition, Yerba Mate aqueous extract was reported to improve plasma lipid profile both in vitro (3T3-L1 cells model) and in vivo (mice model), probably by inhibiting adipogenesis via downregulating the expression of adipogenesis related genes (Creb-1 and C/EBPα) [76].

### 4.5. Regulation of Gut Microbiota

More recently, growing attention has been paid to the effect of tea beverages on gut microbiota. Enhanced probiotic colonization was observed in a broiler chicken model fed with ground Yerba Mate leaf supplement (0.55% inclusion rate) [77]. Moreover, *Ilex kudingcha* extract (400 mg/kg) was demonstrated to change the diet-disrupted gut microbiota composition to normal state and increase their diversity in HFD-fed mice [78]. It was reported that polyphenols from *I. latifolia* played a critical role in establishing the structure of gut microbiota, since dietary polyphenols, especially dicaffeoylquinic acids (diCQAs), exhibited low bioavailability in the upper digestive tract, and reached the colon with an intact form and interacted with the colonic microbiota, contributing to the amelioration of the intestinal flora [79]. In addition, Xie et al. reported that diCQAs from Kudingcha enhanced the diversity of intestinal microbiota in vitro and promoted the generation of short-chain fatty acids (SCFAs) through gut microbiota, which in turn provided nutrients and energy for the optimization of gut microbial profile [9]. Therefore, the interaction between tea consumption and intestinal microbes can further improve the microbial colonization and promote human health.

### 4.6. Anti-Cancer Activity

Although epidemiological studies have reported a correlation between Mate tea consumption and esophageal cancer, it is most likely due to confounding factors, such as high consumption temperature rather than the carcinogenic constituents present [80,81], since any beverages with temperature above 65 °C are “likely carcinogenic to humans” [82]. In fact, the cytotoxic action against diverse cancer cells, such as breast cancer, oral cancer, nasopharyngeal carcinoma, and colon adenocarcinoma cells, was reported in Yaupon Holly leaves [20], Kudingcha extracts [5], and Yerba Mate extracts [83,84], which were tested with concentrations ranging from 10 μg/mL to 1000 μg/mL, and could not only inhibit the viability and proliferation of cancer cells, but also prevent metastasis and promote apoptosis of cancer cells. The presence of characteristic ingredients, mainly chlorogenic acid derivatives, may be responsible for the anti-cancer effects of *Ilex* Kudingcha [39].

Several studies also elucidated the anti-cancer molecular mechanisms. It was reported that caffeoylquinic acids in *Ilex* tea extracts were able to activate the pro-apoptotic factors caspase-3 and caspase-9 in TCA8113 cancer cells, and caspase-8 and caspase-3 in HT-29 human colon cancer cells, accompanied with the decreased expression of the inflammatory mediator NF-*κ*B, which regulates cell proliferation, anti-apoptosis, and cell metastasis [6,85]. Overall, induction of cancer cell apoptosis and suppression of chronic inflammation could be two main mechanisms of the anti-cancer activity of *Ilex* tea.

### 4.7. Cardiovascular Protective Activity

Research on cardiovascular protection is limited, and only reported for *I. paraguariensis* and *I. kudingcha*. Kudingcha extract showed ameliorative effects on blood vessel contractility and blood flow in both rats and rabbits [3]. Kudingcha polysaccharides were also reported to have a protective effect against vascular dysfunction in high fructose-fed mice [86]. Yerba Mate consumption had great potential for reducing intermediate factors for cardiovascular diseases in both animal and human interventional studies [87]. In addition, improved blood viscosity and microcirculation were observed in 142 subjects supplemented with Yerba Mate tea (5 g/day) [88]. Moreover, reduced cardiovascular diseases were observed in 95 postmenopausal women consuming more than 1 L/day of mate infusion [89]. Thus, *Ilex* tea has great potential to be used as a preventive or therapeutic ingredient against cardiovascular diseases.

### 4.8. Anti-Obesity Activity

In recent years, reports have shown that caffeinated beverages from the genus *Ilex*, including Yerba Mate and Kudingcha, can reduce body weight and have great potential to be developed into anti-obesity drugs [78]. *I. latifolia* (0.33% aqueous extract was added to the HFD) showed protective effects against HFD-induced body weight gain in mice, accompanied by decreased adipocyte lipid accumulation and suppressed expression of lipogenic genes in the liver [90]. Furthermore, adipocyte size, adipocyte differentiation, and fat accumulation were also suppressed in obese rats after treatment with *I. paraguariensis* aqueous solution [91,92,93,94]. In addition to direct impact on adipogenesis, the anti-obesity effects of Mate extract were correlated with decreased appetite [95]. Hussein et al. found that chronic administration of Yerba Mate (50 mg/kg) induced elevated levels of the satiety markers glucagon-like peptide 1 (GLP-1) and leptin in high-fat diet-fed mice, leading to appetite-suppression and reduced food intake, thus decreasing body weight (BW) and body mass index [96].

Besides, the anti-obesity activity of Yerba Mate beverages has been validated in clinical trials. A randomized double-blind trial conducted by Kim et al. showed that body fat mass was significantly reduced in obese subjects supplemented with oral Yerba Mate capsules (3 g/day) for twelve weeks [97]. In addition, acute intake of Yerba Mate was confirmed to augment energy expenditure in healthy people [98], and it was interesting that a higher increase in energy expenditure could be induced by ingesting Yerba Mate at cold temperatures (e.g., 3 °C) rather than hot temperatures (e.g., 55 °C), without exerting negative impacts on the cardiovascular system [99]. Thus Yerba Mate appears to have great potential to be developed into an anti-obesity functional food.

### 4.9. Anti-Diabetic Activity

Increasing in vitro and in vivo studies support *I. latifolia* as an effective way to control postprandial hyperglycemia. Kudingcha aqueous extracts (6 mg/mL) could decrease 36% and 50% of the Na^+^-dependent and Na^+^-independent glucose absorption by Caco-2 cells in vitro, respectively [100]. In addition, blood glucose levels in the epinephrine hyperglycemia rat models also returned to normal levels under treatment with 5 or 10 g/kg Kudingcha extracts [3]. This result was in agreement with another in vivo study that oral administration of Yerba Mate (100 mg/kg) aqueous extract for seven weeks decreased blood glucose levels and improved insulin sensitivity in Tsumura Suzuki obese diabetic (TSOD) mice, thus reducing the risk of hyperglycemia [97]. The caffeoylquinic acid (CQA) derivatives derived from *I. latifolia* were further confirmed to play an important role in producing these effects, by means of binding to α-glucosidase via stable hydrogen bonding and hydrophobic interaction, thus reducing blood sugar levels [8]. In addition, clinical trials demonstrated the possibility of *I. paraguariensis* beverages for the prevention of diabetes complication, since long-term *I. paraguariensis* consumption (1 L/day, sixty days) improved glycemic profile and pre-diabetes related conditions (oxidative stress and dyslipidemia) in T2DM and pre-diabetic individuals [47]. Therefore, the consumption of herbal teas prepared from *Ilex* species is likely to be beneficial for the treatment of diabetes.

### 4.10. Neuroprotective Activity

The caffeinated beverages from the genus *Ilex* also show neuroprotective activity [101]. For instance, the exposure of cortical neurons to *I. latifolia* (1–100 μg/mL) was reported to inhibit neuronal death induced by glutamate, hypoxia, and amyloid β protein (Aβ) through suppressing the pathway of apoptosis [102,103]. Additionally, in vivo experiments also demonstrated that *I. latifolia* supplement (25 to 200 mg/kg) significantly inhibited Aβ (25–35)-induced memory impairment in mice and ischemia-induced neurological deficits in rats in a dose-dependent manner [103,104]. Clinical results further revealed its potential to inhibit the development of Parkinson’s disease [105].

### 4.11. Other Health Benefits

In addition to the health benefits mentioned above, Yerba Mate was also reported to improve bone mineral density in postmenopausal women and accelerated the healing of the alveolar socket in rats after tooth extraction [106,107]

## 5. Conclusions

In conclusion, this review summarized the distribution and chemical composition of the caffeinated beverages from the genus *Ilex*, including the large-leaved Kudingcha, Yerba Mate, Yaupon Holly, and Guayusa, along with their potential health benefits, including antioxidant, anti-inflammatory, antibacterial, lipid-reducing, regulation of gut microbiota, anti-cancer, cardiovascular protective, anti-obesity, anti-diabetic, neuroprotection, etc. However, the genus *Ilex* contains about 600 species, most of which still lack detailed investigation. In the future, intensive bioprospecting of the whole range of genetic resources is sure to reveal interesting and useful new compounds and new sources of high levels of known compounds. In addition, further research should aim at designing controlled clinical trials to investigate the effects of long-term consumption of well-characterized *Ilex*-based beverages on human health.

## Figures and Tables

**Table 1:** Distribution of the most commonly consumed species of the genus *Ilex*.

Common Name	Species	Distribution	References
Large-leaved Kudingcha	*I. kudingcha* C.J. Tseng	China: Guangxi; Guangdong; Hainan	[3,25,30]
	*I. latifolia* Thunb.	China: Zhejiang; Jiangsu; Fujian; Anhui; Hainan	[3,6,7,10]
Yerba Mate	*Ilex paraguariensis* A. St.-Hil	South America: Argentina; Brazil; Paraguay; Uruguay	[26,27,31,32,33]
Yaupon Holly	*Ilex vomitoria*	Southeastern United States	[19,21,29,34]
Guayusa	*Ilex guayusa* Loes	South America: Argentina, Southern of Brazil, Paraguay and Uruguay	[22,24,35,36]

**Table 2:** Main phenolic compounds in the genus *Ilex.*

Tea Name	Species	Main Polyphenols	Reference
Large-leaved Kudingcha	*Ilex kudingcha* C. J. Tseng	Neochlorogenic acid	[30,37]
		Chlorogenic acid	
		Cryptochlorogenic acid	
		Protocatechuic acid	[37]
		Caffeic acid	
		Isochlorogenic acid	
		Rutin	
	*I*. *latifolia*	Caffeic acid derivatives	[3]
		Ethyl caffeate	[5]
		3,4-di-*O*-caffeoylquinic acid methyl ester	
		3,5-di-*O*-caffeoylquinic acid methyl ester	
		Chlorogenic acid	
Yerba Mate	*Ilex paraguariensis* A. St.-Hil	Hydroxycinnamic acid derivatives	[18]
		Flavonols	
		3-caffeoylquinic acid	
		5-caffeoylquinic acid	
		4-caffeoylquinic acid	
		3, 5-dicaffeoylquinic acid	
		Rutin	
Yaupon holly	*I*. *vomitoria*	Rutin	[20,21]
		Chlorogenic acid	[21]
		Neochlorogenic acid	
		Cryptochlorogenic acid	
Guayusa	*I*. *guayusa*	Chlorogenic acid	[22,24]
		Isochlorogenic acid	[22]
		Neochlorogenic acid	
